# The usefulness of “corrected” body mass index vs. self-reported body mass index: comparing the population distributions, sensitivity, specificity, and predictive utility of three correction equations using Canadian population-based data

**DOI:** 10.1186/1471-2458-14-430

**Published:** 2014-05-06

**Authors:** Daniel J Dutton, Lindsay McLaren

**Affiliations:** 1 Department of Community Health Sciences, University of Calgary, Calgary, Alberta, Canada

**Keywords:** Body mass index, Measurement error, Obesity, Overweight, Self-report, Bias, Correction

## Abstract

**Background:**

National data on body mass index (BMI), computed from self-reported height and weight, is readily available for many populations including the Canadian population. Because self-reported weight is found to be systematically under-reported, it has been proposed that the bias in self-reported BMI can be corrected using equations derived from data sets which include both self-reported and measured height and weight. Such correction equations have been developed and adopted. We aim to evaluate the usefulness (i.e., distributional similarity; sensitivity and specificity; and predictive utility vis-à-vis disease outcomes) of existing and new correction equations in population-based research.

**Methods:**

The Canadian Community Health Surveys from 2005 and 2008 include both measured and self-reported values of height and weight, which allows for construction and evaluation of correction equations. We focused on adults age 18–65, and compared three correction equations (two correcting weight only, and one correcting BMI) against self-reported and measured BMI. We first compared population distributions of BMI. Second, we compared the sensitivity and specificity of self-reported BMI and corrected BMI against measured BMI. Third, we compared the self-reported and corrected BMI in terms of association with health outcomes using logistic regression.

**Results:**

All corrections outperformed self-report when estimating the full BMI distribution; the weight-only correction outperformed the BMI-only correction for females in the 23–28 kg/m^2^ BMI range. In terms of sensitivity/specificity, when estimating obesity prevalence, corrected values of BMI (from any equation) were superior to self-report. In terms of modelling BMI-disease outcome associations, findings were mixed, with no correction proving consistently superior to self-report.

**Conclusions:**

If researchers are interested in modelling the full population distribution of BMI, or estimating the prevalence of obesity in a population, then a correction of any kind included in this study is recommended. If the researcher is interested in using BMI as a predictor variable for modelling disease, then both self-reported and corrected BMI result in biased estimates of association.

## Background

Obesity’s rise in prevalence over the past 30 years [1], coupled with knowledge of its public health burden [2-6], has opened debate over the best way to measure adiposity in populations. The body mass index - BMI [weight (kg)/height^2^ (m^2^)] - is a common measure in population-based surveys: it is relatively inexpensive, simple, and non-intrusive. Notwithstanding the limitations of BMI as a measure of adiposity [7-10], it continues to be recommended by the World Health Organization [10] as the appropriate criteria to assess obesity status in populations due to the high correlation of high BMI with excess body fat and poor health outcomes [1].

The practice of gathering self-reported height and weight data from survey respondents has raised concerns about the inaccuracy of self-reported data. Where comparison to measured weight is possible, studies have demonstrated misreporting (both under- and over-reporting) of weight, which varies by sex, age, race/ethnicity, and BMI [11-19]. The consequence of this misreporting is that the potential exists for large bias in estimates of prevalence and measures of association in studies that use self-reported BMI [7].

By using population-based datasets which contain both measured and self-reported values of BMI for the same individuals, it is possible to develop statistical adjustments that bring self-reported values of BMI closer to measured values. These correction methods can then be applied to datasets that only contain self-reported height and weight, resulting in a corrected height and weight and thus improving the usability of those datasets. Attempts to develop correction methods for use with Canadian data have resulted in two important papers. Connor Gorber et al. [20] used data from the 2005 Canadian Community Health Survey (CCHS) and, after considering many potential covariates, concluded that the most parsimonious and effective correction equations for men and women rely on one variable: self-reported BMI. Shields et al. [21] used data from the 2007–2009 Canadian Health Measures Survey to develop a correction equation, which was then used to correct self-reported BMI measures from the 2008 CCHS. This second paper likewise concluded that a correction equation using only self-reported BMI is appropriate for correcting BMI values. In both studies, additional covariates did not add enough predictive power to the models to justify the added complexity of including them in the correction equations.

The practice of correcting based on BMI alone (i.e., no other covariates) is appealing in its simplicity. Other authors have included covariates in an effort to increase the accuracy of their correction equations; including age [22], leisure time physical activity, self-reported health [14], education level [23], and ethnicity [24], among others. These studies find that including additional covariates in a correction equation can increase the correction equation’s accuracy when adjusting BMI or categorizing individuals by obesity status (e.g., BMI > 30). In this paper we did not include additional covariates to maximize comparability with the existing Canadian correction equations.

A separate issue is whether corrections should be based on BMI, or weight alone. Studies from the United States [11,13], as well as from Sweden [14], and France [15] verify that average misreporting increases as measured weight and/or BMI increases, suggesting that correcting on either BMI or weight may be acceptable. Correcting on weight only raises the question of whether or how to deal with height. It is well-documented that height is subject to bias in the elderly, i.e., those over age 60 [11,22] and that bias in height has been shown to be substantial [25,26]. Furthermore, international evidence shows that height bias also exists among those under age 60, is strongest for the shortest males and for females, and that bias might be changing over time [23,24,27]. Correction equations developed by other authors have directly corrected for either BMI as a whole [14,20,21,23,26], or height and weight separately and calculated a corrected BMI from those values [12,23,24] to incorporate both weight and height. However, there is evidence to suggest that average bias in self-reported height among adults as a whole is quite small: based on five national surveys conducted in Canada and the United States on males and females aged 18 to 74, the range of average bias in self-reported height ranged from 0.2 cm to 1.4 cm [28]. Because the bias in working age adults is almost wholly from bias in self-reported weight [11-17,26], it is worth considering the value of correcting on weight only rather than overall BMI for the general population. This paper will consider such a correction.

The purpose of this study is to evaluate the usefulness of existing and new correction equations for BMI in population-based research. To accomplish this, we have three objectives: 1) compare the self-reported and corrected BMI distributions; 2) compare self-reported and corrected BMI to measured BMI based on sensitivity and specificity of measured obesity; and 3) compare self-reported and corrected BMI to measured BMI in regression models of various health conditions, in terms of statistical significance, coefficient magnitude, and direction of the coefficient (above or below the measured coefficient). We compare three correction equations: first, an existing Canadian correction equation [20] (a correction that used self-reported BMI, so will be referred to as the “BMI-only” correction); second, a new correction equation developed here which corrects values of weight only; and third, another correction equation developed here which is a computationally simpler version of the weight-only correction. The term “weight-only” means that we use a corrected value for weight but self-reported height to correct overall BMI.

## Methods

### Data

We used data from two cycles (2005 and 2008) of Statistics Canada’s Canadian Community Health Survey (CCHS). The CCHS is a repeated cross-sectional survey that provides socio-demographic and health information for individuals living in the ten Canadian provinces. The CCHS uses a multi-stage cluster sampling procedure to derive a sample that is representative of the Canadian population, excluding those that live in institutions, on First Nations reserves, on Canadian Forces bases, and in certain remote areas; the CCHS is representative of approximately 98% of the Canadian population over age 12 [29]. The overall response rates for households were 87.0% for the 2005 CCHS was and 85% for the 2008 CCHS [21].

In the 2005 and 2008 iterations of the survey, a random sub-sample of individuals was asked to self-report their height and weight; those values were subsequently measured by the interviewer. The respondents were not told they would be measured when they self-reported their height and weight. The response rate for the sub-sample was 64.2% for 2005 CCHS and 59.7% for the 2008 CCHS; no information is available for reason of refusal to be measured [21]. We focused on individuals aged 18 to 65 for whom both self-reported and measured BMI data were available. We excluded adults over age 65, because of observed over-reporting of height in the over 65 age group [11,25,26].

We used the master file versions of the CCHS, accessed through the Research Data Centres (secure data laboratories) program in Canada. Access was granted by Statistics Canada via the Canadian Research Data Centre Network (CRDCN) through a standardized application process. All analyses incorporated sampling weights as directed by Statistics Canada and were conducted in Stata 11.2. Ethics approval for this project was obtained from the University of Calgary’s Conjoint Health Ethics Research Board (Ethics ID: E-23704).

### Procedure

Below, we first describe the development of the weight-only correction equations. Then, we describe the procedure for achieving our two objectives that compare self-reported and corrected BMI to measured BMI. All analyses, including those involved in developing the correction equations, are conducted for males and females separately.

The justification for modelling misreporting based on weight only is best shown graphically. Additional file 1 shows three quantile-quantile plots comparing measured BMI to three other BMI measures: self-reported BMI (graph a); a BMI constructed from self-reported weight and measured height (graph b); and a BMI constructed from measured weight and self-reported height (graph c), for males and females. The graphs show the average BMI for each percentile of measured BMI against the average BMI for each percentile of the BMI measures containing at least one self-reported value. Note that the quantile-quantile plots in graphs a and b look similar, that is, there is very little improvement in modelling measured BMI by replacing self-reported height with measured height. Graph c shows that there is a large improvement in modelling measured BMI using only measured weight, which indicates that the majority of the measurement error of the distribution of self-reported BMI comes from self-reported weight, not height, in our sample. Thus, the weight-only correction should be addressing the main source of measurement error in the sample of working age individuals.

#### a) Development and estimation of weight-only correction equation

We can model the self-reported value of BMI as being a function of an individual’s measured (true) BMI multiplied by a misreporting term:

(1) $$\frac{W_{\text{SR}}}{h_{\text{SR}}^{2}}=\frac{W_{M}}{h_{M}^{2}}\cdot e^{\epsilon +W_{M}}$$

That is, an individual’s self-reported BMI (which is their self-reported weight (*W*_*SR*_) over their self-reported height squared ($h_{\text{SR}}^{2}$) is equal to their measured BMI and a misreporting term that is made up of random noise, *ϵ*, and measured weight *W*_*M*_. Equation (1) was chosen because, with the right parameters^a^, we can mimic the nonlinear relationship between measured and self-reported BMI described in the literature, whereby the discrepancy increases across measured BMI at an increasing rate due to increases in weight. By including weight in the exponential term, we allow the difference between self-reported BMI and measured BMI to grow at an increasing rate as measured weight increases, a relationship that is supported by published literature [11-17]. A linear error term would not accurately capture that association.

Next, we can take the natural logarithm of both sides of the equation to reduce the BMI relationship into its constituent parts, which can then be rearranged into the following equation:

$$\ln\;\left(W_{\text{SR}}\right)=\ln\;\left(W_{M}\right)+W_{M}+2\;\ln\;\left(\frac{h_{\text{SR}}}{h_{M}}\right)+\epsilon$$

where ln(*W*_*SR*_), the natural logarithm of an individual’s weight, is a function of measured weight and the ratio of self-reported to measured height (the relative misreport in height). This is an equation for which we can estimate regression parameters, using a sample of individuals with both measured and self-reported height. The estimated regression equation is:

(2) $$\begin{array}{ll} \;\ln\;\left(W_{\text{SR},i}\right)= & {\hat{\beta }}_{0}+{\hat{\beta }}_{1}\cdot \ln\;\left(W_{M,i}\right)+{\hat{\beta }}_{2}\cdot W_{M,i}\\ & +{\hat{\beta }}_{3}\cdot 2\ln\;\left(\frac{h_{\text{SR},i}}{h_{M,i}}\right) \end{array}$$

Where i denotes individual values. If the ratio of self-reported to measured height is not related to self-reported weight on average (which we would expect from the literature on non-seniors), then ${\hat{\beta }}_{3}$ would be statistically equal to zero, leaving us with the equation:

(3) $$\ln\;\left(W_{\text{SR},i}\right)={\hat{\beta }}_{0}+{\hat{\beta }}_{1}\cdot \;\ln\;\left(W_{M,i}\right)+{\hat{\beta }}_{2}\cdot W_{M,i}$$

The restriction necessary for equation (3) to be an appropriate step, that ${\hat{\beta }}_{3}$ is statistically equal to 0, was tested using our dataset during the model building exercise and is not an assumption. Specifically, using a t-test, we could not reject the null hypothesis that the coefficient ${\hat{\beta }}_{3}$ was equal to 0 (p-value of 0.609 in males and 0.559 in females). Removal of the ratio of self-reported to measured height did not impact the values of the other coefficients in the model.

Equation (3) can be rearranged to put measured weight in terms of self-reported weight and estimated parameters (constants):

(4) $$\ln\;\left(W_{M,i}\right)+\frac{{\hat{\beta }}_{2}}{{\hat{\beta }}_{1}}\cdot W_{M,i}=\frac{\ln\;\left(W_{\text{SR},i}\right)-{\hat{\beta }}_{0}}{{\hat{\beta }}_{1}}$$

One can solve this equation numerically using a dataset that contains both measured and self-reported weight. Using self-reported values, we back-solved for measured weight by iteratively substituting in values for measured weight until the equality held at a predetermined tolerance (number of decimal places, in our case 0.001) to solve for a corrected weight in place of measured weight.

The parameter associated with the measured weight term in misreporting, ${\hat{\beta }}_{2}$ in equation (2), could be quite small in practice. ${\hat{\beta }}_{2}$ could be quite small because it is the coefficient of a variable that is measured in kilograms while the other terms in the equation are measured in the natural log of kilograms. Thus, measured weight, the variable that ${\hat{\beta }}_{2}$ is associated with, need only have a small effect to make a large impact on the natural log of self-reported weight. It might be the case that, for the ranges of BMI exhibited by the majority of the population, ${\hat{\beta }}_{2}$ is essentially zero. To accommodate this possibility, we also consider an alternative form of equation (1):

(5) $$\frac{W_{\text{SR}}}{h_{\text{SR}}^{2}}=\frac{W_{M}}{h_{M}^{2}}\cdot e^{\epsilon }$$

Equation (5) assumes that natural log of self-reported weight depends only the natural log of measured weight and on a constant term that captures the average effect of unobserved variables. This equation leads to a much simpler regression equation:

(6) $$\ln\;\left(W_{\text{SR},i}\right)={\hat{\beta }}_{0}+{\hat{\beta }}_{1}\cdot \ln\;\left(W_{M,i}\right)$$

and correction equation if we follow the same steps as outlined for equation (1):

(7) $$W_{M,i}=e^{\frac{\ln\;\left(W_{\text{SR},i}\right)-{\hat{\beta }}_{0}}{{\hat{\beta }}_{1}}}$$

For the remainder of this paper, equation (4) will be referred to as the “weight-only correction”, and equation (7) as the “simple weight-only correction”. Equation (6) is similar to the equation developed by Connor Gorber et al. [20] in the sense that it does not include other covariates other than the measured and self-reported versions of the variable being corrected. The evaluation process below aims to test correction equations that would be widely usable due to their simplicity. Connor Gorber et al. [20] showed that the inclusion of other covariates such as age, perception of one’s own weight, life dissatisfaction, ethnicity, and activity limitations did not importantly improve the accuracy of their models. Thus, we do not include any other variables in the correction equations to facilitate comparison with the existing recommended Canadian correction equations.

We first defined the full sample and identified outliers. The full sample consists of the pooled cross-section of the 2005 and 2008 CCHS respondents who provided measured and self-reported height and weight (n = 6294: 3208 female, 3086 male), restricted to working-age individuals (age 18 to 65) and non-breastfeeding, non-pregnant women. Outliers (n = 145) were defined as those for whom the discrepancy between self-reported and measured height or weight exceeded three standard deviations from the sex- and cycle-specific mean discrepancy. The identification and removal of outliers served to remove their potentially undue influence during correction generation.

The full sample of non-outliers was split randomly in half. One half, randomly selected, was used to calibrate the weight-only correction model parameters (the “model generating group”, n = 3084). The other half (“test group”, n = 3210) was used to test the model parameters to see how well the adjusted BMI values compared to measured BMI. The previously excluded outliers were included in the test group to simulate a real dataset where outliers may appear to have reasonable values of self-reported height or weight and may be impossible to identify and exclude. The regression equations of interest, (3) and (6), were run on the model generating group for males and females separately to obtain the necessary parameters for the correction equations. Male- and female-specific correction equations were developed separately to allow for known different trends in misreporting weight for males and females [12,13], and to match the convention used in other Canadian corrections [20,21] and international corrections [14,23,24]. This stratification by sex was maintained for all analyses.

The model-generating group consisted of only working age adults (i.e., 18–65), but the correction equations are appropriate to apply to adults over age 65. This was confirmed by a Chow test for equation (3) which was run separately for males and females. The mutually exclusive groups under consideration were 1) working age adults and 2) adults over age 65. The null hypothesis that the models have the same coefficients for both working age adults and adults over age 65 could not be rejected (for males the p-value was 0.167 for females the p-value was 0.292).

After obtaining estimates for the parameters, we applied correction equations (4) and (7) to the test group. This model solves for corrected weight, so to make a corrected value of BMI with the weight-only model we used corrected weight with self-reported height. For the test group, we report weight-only as well as simple weight-only corrections, along with BMI-only corrections using the Connor Gorber et al. model [20].

#### b) Evaluation of correction equations: comparison of BMI distributions; estimation of sensitivity and specificity for weight categories; and prediction of health outcomes

The different weight-only correction equations (regular and simple), which are calibrated versions of equations (4) and (7), for males and females, are given below:

Males (Weight-only Correction):

$$\ln\;\left(W_{M}\right)+\frac{.002}{.788}W_{M}=\frac{\ln\;\left(W_{\text{SR}}\right)-.710}{.788}$$

Females (Weight-only Correction):

$$\ln\;\left(W_{M}\right)+\frac{.001}{.811}W_{M}=\frac{\ln\;\left(W_{\text{SR}}\right)-.697}{.811}$$

Males (Simple weight-only Correction):

$$W_{M}=e^{\left(\frac{\ln\;\left(W_{\text{SR}}\right)-.304}{.926}\right)}$$

Females (Simple weight-only Correction):

$$W_{M}=e^{\left(\frac{\ln\;\left(W_{\text{SR}}\right)-.209}{.942}\right)}$$

We evaluate the correction equations for usefulness in three ways: first, we compare self-reported, corrected, and measured BMI distributions. Second we compare self-reported and corrected BMI to measured BMI by estimating the sensitivity/specificity of the equations vis-a-vis BMI categories of normal weight (18 < BMI < 25), overweight (25 ≤ BMI < 30), and obese (BMI ≥ 30). Lastly, we compare self-reported and corrected BMI to measured BMI by association with selected health outcomes in regression models. We compare the regression results in terms of statistical significance, coefficient magnitude, and direction of the coefficient (above or below the measured coefficient). Assessment of association with health outcomes entailed comparing coefficients (statistical significance, magnitude, and direction) across the different corrected BMI values in regression equations modeling arthritis, heart disease, diabetes, high blood pressure, self-reported health, and activity limitation, following Connor Gorber et al. [20]. We use the BMI categories of normal weight (18 < BMI < 25), overweight (25 ≤ BMI < 30), obese (30 ≤ BMI < 35), and obese class II or higher (BMI ≥ 35) for this part of the analysis. We further restrict this part of the analysis to individuals aged 40 or older, to follow the convention set by Connor Gorber et al. [20]; however we also test the disease association models with the full age range (18–64). Throughout our analysis we do not report results for underweight individuals because there were so few underweight individuals by measured BMI.

## Results

### Distribution of BMI

**Figure 1:**
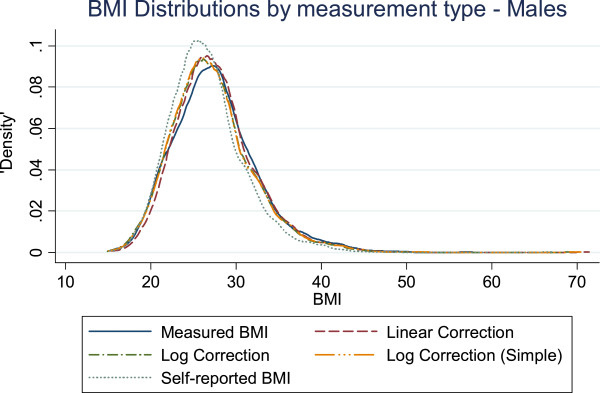
The distribution of BMI as measured by different correction equations and self-report compared to measured BMI in males.

**Figure 2:**
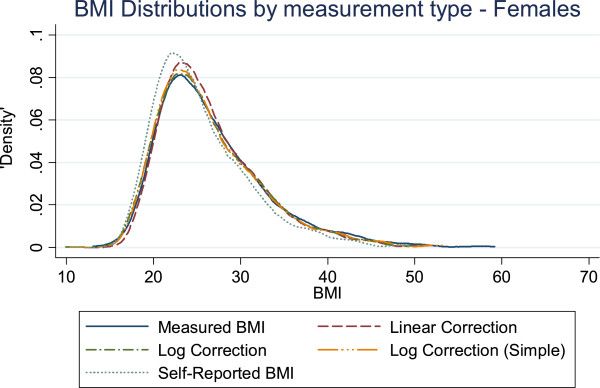
The distribution of BMI as measured by different correction equations and self-report compared to measured BMI in females.

Figures 1 and 2 show the BMI distributions estimated from the measured, self-reported, and corrected BMI values. For males the corrected distributions all trend together, and all are closer to the measured distribution than to the self-reported distribution. For females, the weight-only corrections resemble the measured BMI distribution more closely than the BMI-only correction does, most notably between BMI 23 kg/m^2^ and 28 kg/m^2^.

### Sensitivity and specificity of corrected and self-reported BMI

Table 1 displays the sensitivity and specificity estimates based on the self-reported and corrected values for BMI for males and females. Focusing on those instances with at least a 5 percentage point difference in sensitivity or specificity within sex-BMI category groups, we observed the following patterns: All corrections were similar to one another and superior to self-report in specificity of normal weight among women, sensitivity of overweight among both men and women, and sensitivity of obese among both men and women. There were two instances in which self-report was superior to corrected values: sensitivity of normal weight among both men and women. Overall, any corrected BMI was superior to self-reported BMI in estimating prevalence within weight categories.

**Table 1:** Sensitivity and specificity estimates using self reported BMI and the three correction equations

**MALES – Selected BMI indicators versus measured BMI**						
	**Normal weight**		**Overweight**		**Obese**	
	***Sens***	***Spec***	***Sens***	***Spec***	***Sens***	***Spec***
Self-reported	91.03	83.31	73.03	79.84	60.59	98.52
	(86.21 to 95.85)	(80.16 to 86.45)	(68.29 to 77.78)	(75.40 to 84.28)	(52.91 to 68.27)	(97.56 to 99.48)
BMI Correction	77.11	92.85	82.18	80.02	79.72	94.87
	(70.59 to 83.63)	(90.70 to 95.00)	(77.97 to 86.39)	(75.60 to 84.44)	(73.63 to 85.60)	(92.97 to 96.77)
Weight-only Correction	81.07	89.86	80.15	80.94	75.6	96.82
	(74.62 to 87.52)	(87.20 to 92.51)	(75.82 to 84.48)	(76.40 to 85.49)	(68.79 to 82.42)	(95.48 to 98.16)
Weight-only Corr. (Simple)	81.22	89.86	80.15	80.94	75.6	96.82
	(74.77 to 87.67)	(87.20 to 92.51)	(75.82 to 84.48)	(76.40 to 85.49)	(68.79 to 82.42)	(95.48 to 98.16)
**FEMALES – Selected BMI indicators versus measured BMI**						
	**Normal weight**		**Overweight**		**Obese**	
	***Sens***	***Spec***	***Sens***	***Spec***	***Sens***	***Spec***
Self-reported	91.57	78.66	63.01	88.97	66.44	99.07
	(88.44 to 94.70)	(74.95 to 82.34)	(56.87 to 69.14)	(86.03 to 91.90)	(59.11 to 73.77)	(98.51 to 99.63)
BMI Correction	86.08	87.71	76.88	87.92	81.4	96.55
	(81.83 to 90.34)	(84.88 to 90.55)	(71.62 to 82.15)	(84.88 to 90.97)	(75.65 to 87.15)	(95.12 to 97.98)
Weight-only Correction	86.17	88.96	75.31	89	81.62	96.42
	(82.13 to 90.21)	(86.28 to 91.63)	(69.96 to 80.66)	(86.13 to 91.87)	(75.87 to 87.36)	(94.98 to 97.86)
Weight-only Corr. (Simple)	86.64	88.21	74.13	89.33	81.7	96.42
	(82.66 to 90.62)	(85.46 to 90.96)	(68.69 to 79.56)	(86.50 to 92.16)	(75.96 to 87.44)	(94.98 to 97.86)

95% confidence intervals in parentheses.

### Predictive utility of corrected BMI in health condition models

Tables 2 and 3 show the results of models regressing six health conditions on BMI (measured; self-report; BMI-only correction; weight-only corrections) for men and women, controlling for age. Focusing on differences in statistical significance (i.e., presence/absence) between coefficients in the measured BMI model and coefficients in each of the other models, the following is apparent: For men, of the 18 coefficients in each column, 16 in the self-reported BMI column had the same statistical significance status as measured BMI. The numbers for the BMI-only, weight-only, and simple weight-only corrections were 15/18, 16/18, and 15/18, respectively. For women, of the 18 coefficients in each column, 13 in the self-reported BMI column had the same statistical significance status as measured BMI. The numbers for the BMI-only, weight-only, and simple weight-only corrections were 15/18, 15/18, and 14/18, respectively. From this preliminary consideration of statistical significance, the correction equations and self-report BMI appear to perform similarly for men, while for women the correction equations appear to perform similarly to each other and better than self-reported BMI.

**Table 2:** Odds ratios from regressions for BMI category and selected health conditions for males, controlling for age (N = 821)

	**Measured BMI**		**Self-reported BMI**		**BMI correction**		**Weight-only correction**		**Weight-only correction (simple)**	
**Health condition**	**OR**	**95% CI**	**OR**	**95% CI**	**OR**	**95% CI**	**OR**	**95% CI**	**OR**	**95% CI**
**Arthritis**										
Normal weight	1.00		1.00		1.00		1.00		1.00	
Overweight	0.92	0.35 to 2.46	1.81	0.90 to 3.64	1.78	0.82 to 3.86	1.98	0.93 to 4.23	1.98	0.93 to 4.23
Obese (Class I)	0.98	0.34 to 2.81	2.00	0.86 to 4.67	1.61	0.69 to 3.80	2.07	0.90 to 4.76	2.03	0.87 to 4.74
Obese (Class II+)	1.52	0.43 to 5.33	3.79*	1.40 to 10.28	2.54*	1.00 to 6.41	2.83*	1.09 to 7.33	2.88*	1.15 to 7.23
**Heart disease**										
Normal weight	1.00		1.00		1.00		1.00		1.00	
Overweight	0.84	0.30 to 2.37	1.23	0.46 to 3.35	1.45	0.47 to 4.51	0.97	0.34 to 2.72	0.97	0.34 to 2.72
Obese (Class I)	1.36	0.41 to 4.53	1.42	0.48 to 4.22	1.21	0.35 to 4.17	0.92	0.28 to 3.02	0.96	0.29 to 3.14
Obese (Class II+)	0.79	0.19 to 3.27	1.82	0.40 to 8.20	2.21	0.62 to 7.95	2.02	0.61 to 6.64	1.82	0.56 to 5.94
**Diabetes**										
Normal weight	1.00		1.00		1.00		1.00		1.00	
Overweight	0.99	0.33 to 2.95	0.75	0.29 to 1.93	1.30	0.45 to 3.74	1.32	0.47 to 3.70	1.32	0.47 to 3.70
Obese (Class I)	2.90*	1.02 to 8.22	2.05	0.71 to 5.92	2.28	0.76 to 6.82	2.68	0.93 to 7.71	2.78	0.97 to 8.05
Obese (Class II+)	4.81*	1.48 to 15.64	5.89*	2.04 to 17.01	5.40*	1.76 to 16.52	6.95*	2.34 to 20.67	6.07*	2.06 to 17.88
**High blood pressure**										
Normal Weight	1.00		1.00		1.00		1.00		1.00	
Overweight	1.01	0.50 to 2.04	1.56	0.85 to 2.87	1.28	0.63 to 2.60	1.02	0.53 to 1.96	1.02	0.53 to 1.96
Obese (Class I)	2.98*	1.38 to 6.44	3.46*	1.64 to 7.32	3.30*	1.51 to 7.22	3.40*	1.66 to 6.97	3.47*	1.68 to 7.15
Obese (Class II+)	4.56*	1.81 to 11.50	5.33*	2.04 to 13.95	3.00*	1.19 to 7.54	2.98*	1.21 to 7.39	2.85*	1.18 to 6.88
**Self-reported health (fair or poor)**										
Normal weight	1.00		1.00		1.00		1.00		1.00	
Overweight	0.75	0.35 to 1.63	0.84	0.42 to 1.66	0.61	0.28 to 1.29	0.58	0.28 to 1.23	0.58	0.28 to 1.23
Obese (Class I)	1.26	0.59 to 2.71	1.65	0.79 to 3.47	1.32	0.61 to 2.88	1.54	0.73 to 3.24	1.61	0.76 to 3.39
Obese (Class II+)	1.33	0.48 to 3.71	1.50	0.57 to 3.96	1.55	0.62 to 3.88	1.93	0.79 to 4.74	1.70	0.70 to 4.16
**Activity limitation (often or sometimes)**										
Normal weight	1.00		1.00		1.00		1.00		1.00	
Overweight	1.13	0.66 to 1.93	1.08	0.65 to 1.78	0.95	0.55 to 1.64	0.92	0.54 to 1.56	0.92	0.54 to 1.56
Obese (Class I)	1.53	0.79 to 2.96	1.70	0.90 to 3.24	1.18	0.62 to 2.26	1.35	0.72 to 2.56	1.43	0.75 to 2.70
Obese (Class II+)	2.55*	1.15 to 5.65	3.53*	1.51 to 8.25	1.88	0.83 to 4.25	2.38*	1.03 to 5.54	2.01	0.89 to 4.53

*statistically significantly different from Normal Weight Odds Ratio (p < 0.05).

Models control for age. There were so few observations in the underweight category that those individuals are excluded from this analysis. Regression performed only using individuals age 40 or higher.

**Table 3:** Odds ratios from regressions for BMI category and selected health conditions for females, controlling for age (N = 942)

	**Measured BMI**		**Self-reported BMI**		**BMI correction**		**Weight-only correction**		**Weight-only correction (simple)**	
**Health condition**	**OR**	**95% CI**	**OR**	**95% CI**	**OR**	**95% C**I	**OR**	**95% CI**	**OR**	**95% CI**
**Arthritis**										
Normal weight	1.00		1.00		1.00		1.00		1.00	
Overweight	1.08	0.61 to 1.90	1.04	0.61 to 1.77	1.21	0.69 to 2.11	1.22	0.70 to 2.13	1.02	0.58 to 1.77
Obese (Class I)	1.24	0.66 to 2.34	2.72*	1.56 to 4.74	1.67	0.94 to 2.97	1.70*	0.96 to 3.00	1.54	0.87 to 2.74
Obese (Class II+)	4.97*	2.45 to 10.08	4.29*	1.85 to 9.97	5.04*	2.45 to 10.37	4.92*	2.41 to 10.04	4.36*	2.16 to 8.79
**Heart disease**										
Normal weight	1.00		1.00		1.00		1.00		1.00	
Overweight	0.47	0.10 to 2.23	2.50	0.69 to 8.96	1.78	0.48 to 6.65	1.90	0.51 to 7.09	1.97	0.53 to 7.33
Obese (Class I)	1.45	0.34 to 6.16	2.64	0.96 to 7.22	1.63	0.55 to 4.84	1.64	0.54 to 4.95	1.69	0.56 to 5.10
Obese (Class II+)	2.04	0.39 to 10.64	4.54	0.83 to 24.67	3.29	0.82 to 13.14	3.44	0.88 to 13.55	3.43	0.87 to 13.47
**Diabetes**										
Normal weight	1.00		1.00		1.00		1.00		1.00	
Overweight	0.71	0.21 to 2.46	1.43	0.41 to 4.94	6.28*	1.67 to 23.56	4.30*	1.25 to 14.77	4.45*	1.29 to 15.32
Obese (Class I)	0.90	0.24 to 3.33	1.85	0.65 to 5.26	3.51*	1.01 to 12.13	2.53	0.83 to 7.71	2.42	0.87 to 7.51
Obese (Class II+)	3.13	0.78 to 12.44	5.26*	1.23 to 22.36	14.56*	3.79 to 55.93	10.29*	3.01 to 35.25	10.55*	3.12 to 35.60
**High blood pressure**										
Normal weight	1.00		1.00		1.00		1.00		1.00	
Overweight	1.45	0.70 to 3.01	2.12*	1.12 to 4.00	1.84	0.92 to 3.67	1.94	0.97 to 3.85	2.00*	1.01 to 3.99
Obese (Class I)	2.26*	1.09 to 4.69	3.70*	1.99 to 6.89	3.19*	1.65 to 6.13	3.27*	1.70 to 6.28	3.38*	1.75 to 6.50
Obese (Class II+)	5.28*	2.20 to 12.68	7.15*	2.57 to 19.90	5.50*	2.27 to 13.27	5.54*	2.32 to 13.26	5.55*	2.35 to 13.09
**Self-reported health (fair or poor)**										
Normal weight	1.00		1.00		1.00		1.00		1.00	
Overweight	0.70	0.34 to 1.44	1.05	0.51 to 2.17	0.95	0.46 to 1.95	0.98	0.47 to 2.01	1.02	0.49 to 2.10
Obese (Class I)	1.19	0.54 to 2.62	2.25*	1.12 to 4.53	1.31	0.63 to 2.69	1.34	0.65 to 2.75	1.18	0.59 to 2.37
Obese (Class II+)	2.29	0.84 to 6.22	2.55	0.69 to 9.30	2.37	0.86 to 6.50	2.37	0.87 to 6.46	2.70*	1.03 to 7.12
**Activity limitation (often or sometimes)**										
Normal weight	1.00		1.00		1.00		1.00		1.00	
Overweight	1.23	0.75 to 2.02	1.31	0.79 to 2.19	0.96	0.58 to 1.57	0.97	0.59 to 1.58	0.90	0.55 to 1.47
Obese (Class I)	1.81	0.96 to 3.43	2.03*	1.15 to 3.57	1.74	0.93 to 3.23	1.74	0.94 to 3.24	1.57	0.84 to 2.94
Obese (Class II+)	3.80*	1.90 to 7.62	3.70*	1.50 to 9.13	3.20*	1.49 to 6.91	3.19*	1.50 to 6.82	3.30*	1.56 to 6.98

*statistically significantly different from Normal Weight Odds Ratio (p < 0.05).

Models control for age. There were so few observations in the underweight category that those individuals are excluded from this analysis. Regression performed only using individuals age 40 or higher.

In terms of the magnitude and direction of the coefficients for the relationships between BMI (self-reported and corrected) and disease outcomes (Tables 2 and 3), the self-reported and corrected BMI measures did not exhibit a consistent pattern for males or females across diseases. For males, the corrected odds ratios were higher than the measured odds ratios in almost all comparisons; exceptions were heart disease and diabetes (obese class I), high blood pressure (obese class II+), and self-reported health (overweight). All of the corrected odds ratios for activity limitation were lower than the measured estimates. For females, the corrected odds ratios are higher than the measured odds ratios for heart disease, diabetes, high blood pressure, and self-reported health (except for the simple weight-only correction for obese class I). The corrected odds ratios for activity limitation are all lower than the measured odds ratios, and for arthritis the weight-only corrections were lower than the measured odds ratios for overweight (simple correction only) and obese class II+. Thus, the magnitudes and directions of the estimated coefficients in the health condition models do not clearly point to a superior correction equation^b^.

Furthermore with respect to estimates in Tables 2 and 3, the corrected odds ratios tended to have wider confidence intervals (suggesting less precision of estimate) than the corresponding measured odds ratios, but not in every case. Considering only obese class II + as an example, for males the corrected confidence intervals were wider than the measured confidence intervals for arthritis, heart disease, diabetes, and self-reported health. For females the corrected confidence intervals were wider than the measured confidence intervals in all cases but one (simple weight-only correction for arthritis).

## Discussion

### Distribution of BMI

A corrected BMI distribution, regardless of whether BMI-only or weight-only, was found to be more accurate than the self-reported BMI distribution (see Figures 1 and 2). However, this only refers to the ability of the correction equations to bring the distribution of self-reported BMI into line with the distribution of measured BMI, unconditional on any other variables or restrictions. In terms of which correction is better, for males, it is not obvious that there is a superior corrected distribution. For females, the weight-only corrections follow the measured BMI distribution more closely than the BMI-only correction, making the weight-only corrections the best overall candidate correction for simply constructing the BMI distribution.

### Sensitivity and specificity

Findings from the sensitivity and specificity analyses were mixed, with the weight-only and BMI-only performing similarly well in some cases (specificity for normal weight women, sensitivity for overweight men and women, and sensitivity for obese men and women), and self-report best in others (sensitivity for normal weight men and women). The high sensitivity for self-reported BMI in the normal categories for females is consistent with past observations that normal BMI females, on average, under- or over-report weight to a lower degree than other BMI groups [20,21]. In the absence of a superior correction equation, researchers must trade-off sensitivity and specificity when choosing a correction equation. The outcome of these trade-offs depend on the situation: for example, if a researcher were interested in studying a sample of predominantly normal weight males, where the weight-only corrections result in losing 10 percentage points in sensitivity in exchange for 6 percentage points in specificity, it is not clear that a weight-only correction is superior to self-reported BMI. Simply stating that one percentage point gain offsets another is inappropriate, especially when specificity may be more important for conditions like obesity, where the majority of the population is not obese [7].

### Predictive utility of corrected BMI in health condition models

Taking the population distribution and the sensitivity/specificity findings as a whole, our results suggest that if a researcher is interested in BMI statistics across a population, including estimating prevalence within weight categories, any correction presented here will be preferable to self-reported BMI. However, findings from the analysis of associations with health outcomes tell a different story: namely, findings were mixed, such that no correction was uniformly superior and in some cases the self-reported data outperformed the corrected data. None of the correction equations were able to consistently provide coefficient estimates closer to the measured BMI estimates than those provided by the self-reported estimates. The implication of this finding is that in a disease modelling context correction equations are not necessarily more useful than self-reported BMI.

Our findings differ from other Canadian correction equation studies that have showed the BMI-only correction consistently provides coefficient estimates closer to the measured values [20,21]. In statistics, it is recognized that including a variable exhibiting measurement error on the right hand side of the regression equation can provide a biased estimate of effect [30]. The magnitude of this bias depends on the variance of the mis-measured variable (in our case, measured BMI) and the variance of the measurement error itself (in our case, the difference between measured and self-reported BMI). While the corrections presented here adequately correct the average BMI at percentiles of interest and can be used to estimate the distribution of BMI or the prevalence of obesity, they are unable to correct the variance of self-reported BMI. As a consequence, corrected BMI measures provide biased and inconsistent estimates of association when used as regressors, just as any mis-measured variable would. The key issue in our case is whether the magnitude of the bias is substantial (i.e., clinically or socially significant). We suggest that the magnitude of this bias is too large, and the direction of the bias too unpredictable, for corrected BMI variables to be used in this context of modelling health outcomes.

The health outcome association analysis shows that, given a disease in a logit model and a categorical BMI variable (a very common modelling convention), researchers should not necessarily use a correction equation in an effort to improve self-reported BMI. Further, if a researcher, seeking a conservative range of estimates, chose to report both a corrected and a self-reported estimate, it is not clear that the difference between the two should be meaningful anyway in this regression framework: when the corrected estimate is larger than the self-reported coefficient, it does not conform to the idea that the self-reported BMI estimate serves as an upper bound; when the corrected estimate is smaller than the self-reported coefficient, it is not a guarantee that the corrected estimate is closer to the measured BMI estimate or even on the correct side of the null value. Resorting back to self-reported BMI is going to provide biased estimates of association between BMI and the dependent variable, as well as the biased and inconsistent estimates of association for all the other regressor variables in the model [30]. In short, our results suggest that if a researcher is interested in using BMI as a predictor variable for modelling disease, then both self-reported and corrected BMI result in biased estimates of association.

### Limitations

It is important to acknowledge that, because correction equations are based on particular populations, which change over time and place, the equations themselves are likewise somewhat time and place dependent and should be updated over time. Changing misreporting patterns over time have been shown using data from the United States [27], and Ireland [31] and may reflect, in part, changing social attitudes about obesity. The fact that correction equations can change across time is important for modelling the BMI distribution, but updating a correction equation will not fix the issue of a corrected BMI providing biased and inconsistent regression results unless the update somehow deals with the variance of the misreported variable.

Although the response rate for the CCHS surveys as a whole were reasonably high (87.0% for the 2005 CCHS was and 85% for the 2008 CCHS), another limitation of the study is the lower response rate for the subsamples among which both self-reported and measured data were available and which constituted the basis for this study. The overall response rate for the subsample that provided measured height and weight was 55.9% for 2005 CCHS and 50.7% for the 2008 CCHS, most of the non-response was from refusal to be measured [21]. It is unlikely that those who refuse to be measured are randomly distributed across the BMI distribution, so if the individuals who are heavier are refusing to be measured, any correction equation based on this dataset will be inaccurate, including others that have been developed [20,21]. Strategies to improve response rate across the population are thus desirable.

## Conclusions

The BMI-only correction has been applied to Canadian data extensively (e.g., Orpana et al. [32], Janssen et al. [33], and Barberio and McLaren [34]), attesting to the value of such corrections in the literature. Our findings support the use of BMI-only corrections if the researcher is interested in reporting the distribution of BMI, the prevalence of those above and below the obesity threshold of BMI 30 kg/m^2^, or any other cut-point. On the other hand, if the researcher is interested in estimating the effect of BMI on a health condition, then our findings suggest that corrected BMI, using any of the methods examined here, does not represent an improvement over self-report data.

## Endnotes

^a^“The right parameters” implies that the correct equation would not have a coefficient of 1 on every term, as it stands in equation (1). For instance, a data generating process with the coefficients 0.01 on the measured weight term in the error and 0.01 on the noise term would generate an exaggerated version of the relationship we observe in the literature. So it is just a matter of finding the right parameters to match the data.

^b^The disease models were repeated for the entire sample (age 18 to 65). The results of those models (not reported) are consistent with those from the truncated sample; namely, they do not clearly point to any correction equation being superior.

## Abbreviations

BMI: Body mass index; CCHS: Canadian Community Health Survey.

## Competing interests

The authors declare that they have no competing interests.

## Authors’ contributions

DJD conceptualized the study and led the analysis. LM contributed to study conceptualization and interpretation of results. DJD and LM contributed equally to the writing of the paper. Both authors read and approved the final draft.

## Pre-publication history

The pre-publication history for this paper can be accessed here:

http://www.biomedcentral.com/1471-2458/14/430/prepub

## Supplementary Material

Additional file 1 Quantile-quantile plots comparing measured BMI with BMI constructed from combinations of measured and self-reported height and weight. Click here for file
