# Supplementary material for: The usefulness of “corrected” body mass index vs. self-reported body mass index: comparing the population distributions, sensitivity, specificity, and predictive utility of three correction equations using Canadian population-based data
Source: BMC Public Health. 2014 May 6;14:430. doi: 10.1186/1471-2458-14-430 (PMC4108015; doi:10.1186/1471-2458-14-430)
Supplement: Additional file 1 — Quantile-quantile plots comparing measured BMI with BMI constructed from combinations of measured and self-reported height and weight. [file 1471-2458-14-430-S1.docx]

**Additional File 1 - Quantile-quantile plots comparing measured BMI with BMI constructed from combinations of measured and self-reported height and weight.**

**Graph a: Graph b: Graph c:**
